# Risk prediction models for head and neck cancer: A rapid review

**DOI:** 10.1002/lio2.982

**Published:** 2022-11-28

**Authors:** Craig D. L. Smith, Alex D. McMahon, Alastair Ross, Gareth J. Inman, David I. Conway

**Affiliations:** ^1^ School of Medicine, Dentistry, and Nursing University of Glasgow Glasgow UK; ^2^ Institute of Cancer Sciences University of Glasgow Glasgow UK; ^3^ Cancer Research UK Beatson Institute Glasgow UK

**Keywords:** head and neck cancer, review, risk, risk assessment, risk model

## Abstract

**Background:**

Cancer risk assessment models are used to support prevention and early detection. However, few models have been developed for head and neck cancer (HNC).

**Methods:**

A rapid review of Embase and MEDLINE identified *n* = 3045 articles. Following dual screening, *n* = 14 studies were included. Quality appraisal using the PROBAST (risk of bias) instrument was conducted, and a narrative synthesis was performed to identify the best performing models in terms of risk factors and designs.

**Results:**

Six of the 14 models were assessed as “high” quality. Of these, three had high predictive performance achieving area under curve values over 0.8 (0.87–0.89). The common features of these models were their inclusion of predictors carefully tailored to the target population/anatomical subsite and development with external validation.

**Conclusions:**

Some existing models do possess the potential to identify and stratify those at risk of HNC but there is scope for improvement.

## Graphical abstract

Cancer risk assessment models are used to support prevention and early detection. A rapid review of Embase and MEDLINE identified 14 models, of which six were assessed as “high” quality. Some existing models do possess the potential to identify and stratify those at risk of head and neck cancer but there is scope form improvement.

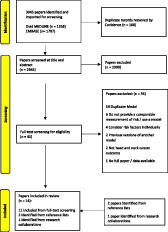

## 1 INTRODUCTION

Head and neck squamous cell carcinomas—generally defined as aerodigestive squamous cancers of the oral cavity, larynx, and pharynx—are a growing challenge for healthcare systems across the world: they are the eighth most common cancer, accounting for an estimated 878,348 new cases and 444,347 deaths globally in 2020.^1^, ^2^ The risk profile of head and neck cancer (HNC) is also changing—with oropharyngeal cancer increasingly associated with human papillomavirus (HPV) infection,^3^ and inequalities in the burden of HNC associated with socioeconomic status.^4^

Overall HNC survival varies greatly by subsite and stage of diagnosis. Despite advancement in treatments, 5‐year survival has seen no major improvements observed in recent decades.^5^, ^6^, ^7^ Furthermore, marginal improvements in prognosis may be undercut by the overall increased disease burden, particularly due to the changing epidemiology of HPV‐associated oropharyngeal cancer.^8^, ^9^ As with all cancers, prognosis is worse with advanced stage disease at presentation. Thus, a major challenge posed by HNC is its traditionally late presentation with over half of cases diagnosed at stage III or IV, when locally advanced or regional or distant metastases are present.^10^, ^11^, ^12^, ^13^, ^14^

Given the twin challenges of increasing HNC incidence and poor survival associated with late‐stage detection, further attention needs to be given to primary and secondary prevention strategies—utilizing the potential of head and neck risk prediction models to identify those at risk and direct them to appropriate prevention and early detection/diagnosis and treatment pathways.

There has already been some success and clinical adoption of other cancer prediction models in primary care; for example, the Q‐series risk prediction models or cancer Risk Assessment Tool.^15^, ^16^ These models have been well evaluated, demonstrating the potential of “personalized medicine” to identify and stratify those at risk.^17^, ^18^, ^19^, ^20^, ^21^ However, they do not assess for HNC risk and there seem to be few risk prediction models for HNC developed or adopted for clinical use. Furthermore, there have been no comprehensive reviews of head and neck risk cancer prediction models or tools published. The aim of this study is to undertake such a review—via systematically searching and identifying models in the international literature, describing their characteristics and performance, quality appraising these models, and performing a narrative synthesis to compare and contrast risk prediction models for HNC.

## 2 METHODS

A rapid review methodology was employed, following the Preferred Reporting Items for Systematic Reviews and Meta‐Analysis (PRISMA) guidelines.^22^ The review was also based on similar reviews on risk prediction models of other cancer sites/diseases.

### 2.1 Search strategy

An electronic literature search of Ovid MEDLINE(R), (and In‐Process, In‐Data‐Review & Other Non‐Indexed Citations), and Embase (1947‐Present, updated daily) databases was conducted using a combination of key headings and search terms associated with “head and neck cancer” and “risk/risk factor/risk assessment” and “prediction/model/tool/score” (see Supplementary Material file for the full list of search terms).

Studies were included if they satisfied all of the following criteria: (i) used a statistical model/tool to predict HNC risk including subsites and potentially malignant conditions; (ii) were published in English; (iii) considered multiple different risk factors; (iv) provided a measurement of risk; and (v) were applicable to the general population.

Given that the focus of this review was on risk prediction, studies that developed prognostic or recurrence models were excluded. Similarly, studies that only considered highly selected groups or risk variables such as highly specific genes were also excluded (as per [v] of exclusion criteria). If multiple publications of the same model were identified, the most extensive and recent report of the model was included.

The reasoning behind a statistical model/tool forming a part of the inclusion criteria was to ensure that there was a robust methodology underlying model development. Crucially, this was also to separate risk models from numerous case–control studies that considered multiple risk factors individually, often expressing these in odds ratios but not evaluating these in the form or context of a risk prediction model or tool. Reporting of a comparable measure of risk was also considered to be, at least, reporting of performance metrics (e.g., AUC) but ideally assigning a value to an individual (e.g., 5‐year risk).

Articles that were ultimately sourced from search, were loaded into Endnote X9 (Clarivate Analytics) reference management software and from here were imported into Covidence (Covidence systematic review software, Veritas Health Innovation), which was used to remove duplicates and perform study screening and data extraction.

### 2.2 Screening and study selection

Two reviewers (CS, DIC) independently screened search results at a title/abstract level and then at a full‐text level using the eligibility criteria. In the event of a disagreement, articles were discussed and included or excluded by mutual consensus.

### 2.3 Data extraction and quality assessment

Following full‐text screening, data extraction was undertaken by two reviewers (CS, DIC) using a customized form containing pre‐defined fields including items on: study characteristics (location, study design, cancer site/subsite, and risk factors included). The data extraction form also assessed the requirement of clinician input (based on whether reported or the nature of the data required to run the model—for instance a patient would not be able to use machine learning tools or conduct HPV serology analysis), along with items on predictive performance (discrimination, sensitivity/specificity, calibration, positive predictive value/negative predictive value [PPV/NPV] and risk threshold cut‐offs) and the method of validation (if undertaken). Measures of discrimination were considered to be “acceptable” if an area under the curve (AUC) value over 0.7 was reported, and “excellent” if a value greater than 0.8 was reported.^23^ Measures of calibration were assessed by the expected/observed ratio or gradient of a calibration slope to the ideal value of 1 and of its intercept to the value of 0.^24^, ^25^

Two reviewers (CS, DIC) also examined the risk of bias of each model using PROBAST, a tool specifically designed to appraise clinical risk prediction models.^26^ Risk of bias (“high,” “low,” or “unclear”) and applicability of the risk models was assessed using 20 questions across four domains (participants, predictors, analysis, and outcomes).

An overall quality assessment was also given to each model considering model validation, and the risk of bias, and applicability concerns assessment (from PROBAST). This was categorized as “High,” “Moderate,” or “Low.” If the model had a (i) low risk of bias, (ii) a low or unclear applicability concern, and (iii) a robust method of validation then it was considered “High” overall quality. Model performance was also considered separately by evaluating each model's discriminative ability. If a model achieved an “excellent” AUC over 0.8 it was classed as high performing (green in table).^27^ Models that achieved acceptable discrimination between 0.7 and 0.8 were classed as moderate (amber in table) and discrimination less than 0.7 was classed as poor (red in table). These classifications also reflected for confidence intervals for model AUC (where reported).

### 2.4 Synthesis

The heterogeneous nature of risk prediction models makes the possibility of pooling the data between the models inappropriate. However, a narrative synthesis was conducted—focusing on the model overall quality/performance and including comparing and contrasting risk factors used in the risk prediction models—grouping them as sociodemographic factors (e.g., age, sex, socioeconomic characteristics), behaviors (smoking, alcohol), biomarkers (e.g., HPV, genetic/polygenic data), clinical information (e.g., symptoms, oral potentially malignant disorders). Models were also compared across subsites of HNC.

## 3 RESULTS

**FIGURE 1:**
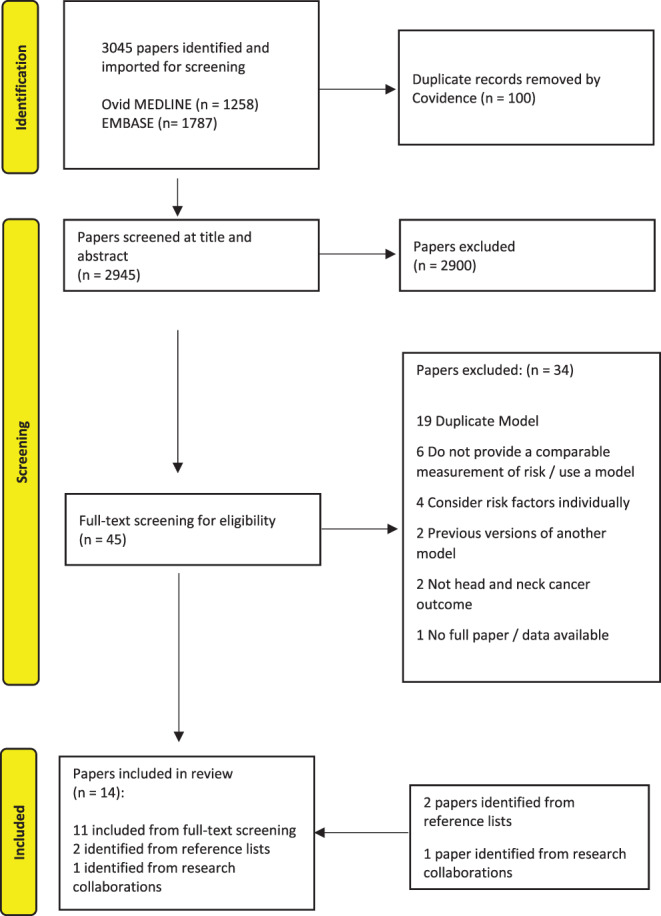
Preferred Reporting Items for Systematic Reviews and Meta‐Analysis flow diagram

Following the removal of 100 duplicates, 2945 studies were identified by the search. Of these, 2900 were excluded by title or abstract screening. Of the remaining 45 studies, 34 were excluded following full‐text assessment, with reasons for exclusion noted (Figure 1). The most common reasons for exclusion were studies did not use a statistical method to develop a risk model, or did not consider multiple risk factors together, or on further examination were a duplication of model already included. One conference abstract paper was excluded because the full text was not available despite attempts to contact the author.

A further three articles were identified—two of these were identified from reviewing the reference lists and the third (at the time of writing yet to be published) was identified from one of reviewer's research collaborations (DIC). Thus, in total, 14 papers were ultimately included in this review (Figure 1).^28^, ^29^, ^30^, ^31^, ^32^, ^33^, ^34^, ^35^, ^36^, ^37^, ^38^, ^39^, ^40^, ^41^ Within these studies, three of the 14 models featured “sub‐models,” using broadly similar methods but stratifying models by subsite or sex.^29^, ^30^, ^35^ These have been reported and considered accordingly, where reported.

### 3.1 Study/model characteristics

A summary of the 14 studies including model characteristics and performance information is shown in Tables 1 and 2 respectively. Of the 14 models, three were conducted in India, one in Sri Lanka, three in China, three in the USA, three in the UK, and one in multiple centers across Northern America/Europe. The majority of the studies (11 out of 14) used a case–control design or built upon previous case–controls for model development. These were mostly of a hospital‐based case–control design. One study used data collected from a randomized control trial.^31^ The other two used a cross‐sectional and prospective cohort study method respectively. There were 11 models which utilized a form of logistic regression analysis approach to evaluating HNC risk. Two of the three remaining models used machine learning methods, while one used a cox‐regression approach to evaluate risk.

**TABLE 1:** Model characteristics

Study ID	Study characteristics		Components of model		
	Country/year/study design	Participants	Cancer outcome/site + sub models	Method + factors included in model	Clinician input
Amarasinghe et al. (2010)	Sri Lanka/Community‐based case–control November 2006–November 2007	101 OPMD cases and 728 controls (men and women)	OPMD excluding lichen planus	Multivariate logistic regression was used to develop the model. Effect estimates of each factor on the risk of OPMD were derived. Gradients for each factor were given a score derived from adjusted odds ratio and ultimately used to develop a 12‐point score cut off model. Predictors used included age, socioeconomic status, betel‐quid chewing, alcohol drinking, smoking.	No
Budhathoki et al. (Unpublished)	North America/Europe—four case–control studies, one prospective cohort—a mixture of hospital and population‐based case–controls. Recruitment dates—not specified	10,126 head and neck cancer cases and 5254 controls	Head and neck cancer (10,126), oral cavity cancer (2431), and oropharyngeal cancer (3727) Six sub models for three sites including separate models for men and women	Predictors were selected based on those that were statistically significant from univariate logistic regression via backwards stepwise selection. The final models used multivariate logistic regression to assess risk with separate models for men and women. Predictors include smoking status, drinking status, education, HPV serostatus and polygenic risk score	Yes
Chen et al. (2018)	China—Hospital based case–control Conducted from September 2010 to March 2017	978 cases and 2646 controls SEPARATE models—1924 men and 1700 women.	Oral cancer (tongue, buccal, gingiva, floor of mouth, palate, lip, and unspecified or overlapping) (380 tongue, 135 buccal, 128 gingival, 72 floor of mouth, 69 palate, 34 lip, and 160 unspecified or overlapping)	Unconditional logistic regression was used, independently significant variables being included in the final nomogram models. Different sets of predictors were used to create separate nomograms for males and females. Men; (smoking [pack‐year], alcohol drinking, tea consumption, fish, seafood, vegetables, fruits, teeth loss, regular dental visits, repetitive dental ulcer) Women; (passive smoking, cooking oil fume exposure, tea consumption, vegetables, fruits, beans, teeth loss, regular dental visits, repetitive dental ulcer, age of first intercourse)	No
Cheung et al. (2021)	India—Analysis of a previous Oral Cancer Randomized Control Trial conducted Screening was conducted in three waves with a 3 year interval between each induvial screening—1996–1998, 1999–2001, and 2002–2004	95,354 control arm 96,516 screening arm (male and female)	Oral cancer	Model was developed using a Cox regression‐based risk prediction model for 7‐year oral cancer incidence. Follow up time was used for a time scale and covariates were selected prior to analysis. Predictors included sex, age, education, BMI, tobacco chewing, tobacco smoking, chewing‐smoking interaction, and alcohol use.	No
Koyanagi et al. (2017)	Japan—Hospital‐based case control 2001—December 2005 365 HNC cases and 1260 controls (male and female)	365 HNC cases and 1260 controls (male and female)	Three separate models—Head and neck cancer (model of interest), upper aerodigestive tract cancer, esophageal cancer	Three models were constructed for each subsite: a genetic, an environmental and an inclusive model. The latter, categories of alcohol and ALDH2 were coded as dummy variables. Interaction terms were used to assess the combined impact of alcohol and ALDH2 interaction. The models were derived using conditional logistic regression models. Predictors included age, sex, ALDH2 genotype, cumulative smoking and alcohol consumption.	Yes
Krishna Rao et al. (2016)	India—Hospital based, unmatched case–control Conducted between July 2011 and August 2012. 180 cases and 272 controls (men and women)	180 cases and 272 controls (men and women)	Oral cavity and oropharynx cancer	Multivariable logistic regression was used to identify significant predictors. Those predictors were included in the final model. This and ROCs were used to develop the risk score and cut offs required for further referral. Predictors included—smoking, chewing tobacco, chewing quid with tobacco, alcohol, spiciness of food, fruit consumption, family history of UADT cancer, rinsing mouth with water after eating/chewing.	No
Lau et al. (2018)	UK—two hospital‐based case–control studies (01/07/2009–01/07/2010 [622 patients]) (1/4/2013–31/8/2013 [453 patients]) 73 cases and 932 controls (men and women)	73 cases and 932 controls (men and women)	Head and neck cancer	Regularized logistic regression was performed on 60% of the data to identify key predictors. Using information form this, the model was refined and underwent split sample (20%) and cross validation (20%). Predictors include age, sex, smoking, alcohol, and symptoms.	Yes
Lee et al. (2020)	USA—Pooled analysis of 14 US‐case control studies from INHANCE consortium. Conducted between 1981 and 2010	7299 HNC cases and 10,301 controls (male and female)	Cancer of the oral cavity, oropharynx, hypopharynx, or larynx. (by subsite) 2388 oral cavity, 2820 oropharynx, 459 hypopharynx, and 1632 larynx (separate models for men and women considering four different subsites—eight sub models)	Logistic Regression Models developed using 70% of the dataset. Hazard and incidence rates from the Surveillance, Epidemiology, and End Results (SEER) Program were applied to the model. Competing risk models were used to ascertain risk by individual subsite but also an overall measure of absolute HNC risk. Models considers age, sex, race/ethnicity, education, cigarette smoking duration and intensity, and/or alcohol drinking intensity; the second set of models additionally included family history of HNC, except for oropharyngeal cancer in both sexes and laryngeal cancer in men.	No
Liu et al. (2017)	China—Hospital‐based Cohort study March 2008 to July 2016.	28 OLK, 41 OSCC, and 18 controls (men and women)	Risk of developing oral cancer from oral leukoplakia	Peaks RF method was used for model development. Split sample testing was used to evaluate the model in addition to 10‐fold cross validation. Subsequently the training set was used to train the model which was used to test the validation set. Predictors included age, sex, site, smoking and drinking.	Yes
McCarthy et al. (2020)	UK—Nested case–control conducted in the UK Biobank 2006–2016	702 HNC cases and 423,050 controls (men and women)	Head and neck cancer excluding laryngeal cancer—no reporting of numbers by subsite	The model was developed using multivariable logistic regression, final predictors being selected upon clinical significance in literature and consultation with a patient and public involvement group Predictors included age, sex, smoking status, townsend deprivation index, body mass index, alcohol consumption, moderate exercise and fruit and vegetable intake.	No
Sharma et al. (2015)	India—“retrospective chart review”—case control June 2004 to June 2009	1025 patients—not specified if men and women	Diagnosis of oral cavity cancer—(no reporting of numbers of cases by subsite)	Dataset underwent filter reduction to select attributes for a PNN/GRNN model—Probabilistic and General Regression Neural Network. A leave one out method was then subsequently used for internal cross‐validation. Attributes included sex, socioeconomic status, clinical symptom, history of addiction, comorbid condition, gross examination, site, predisposing factor, neck nodes, and tumor size.	Yes
Sun et al. (2019)	China—cross‐sectional study Conducted from August 2016 to May 2018	269 patients: OPMD (*n* = 192) and OSCC (*n* = 77) (men and women)	Risk of developing oral cancer from OPMD (leukoplakia or oral lichen planus)	Risk factors included in the final model were developed from univariate logistic regression. Multivariate logistic regression was then used to evaluate the risk factors in a model, the beta‐coefficient being used to assign a risk score to each factor. Cut‐offs were determined using the ROC curve considering sensitivity and specificity. Risk factors included gender, age, lesion site, local stimulus, and alcohol drinking	Yes
Tikka et al. (2020) (v.2)	UK—Prospective data collection, building upon previous version of model developed using case–control. January 2017 until December 2018	307 HNC cases and 3224 controls (men and women)	Head and Neck cancer—“all primary cancers to the HaN regions (*n* = 247), metastatic cancers to the HaN from other regions, including lymphoma (*n* = 48) and cancers in neighboring regions that manifested with HaN symptoms (*n* = 12)”	The final multivariate logistic regression was developed using univariate logistic regression and backwards elimination of non‐statistically significant variables. The final model was then used for bootstrap validation on the final model. Variables included age, gender, unintentional weight loss, smoking, alcohol, positive and negative symptoms/signs of HNC.	Yes
Tota et al. (2019)	USA—Synthetic case–control study	241 Cases (unweighted) and 9327 controls (unweighted) 12,656 vs 154,532,508 (weighted)—men and women	Oropharyngeal cancer	The model was developed using multivariable Weighted binary logistic regression. Cases and controls were propensity weighted according to incidence rates in the population identified by National Cancer Institute's Surveillance, Epidemiology, and End Results (SEER) program. Using this, a 1‐year absolute risk was calculated. The model predictors include age, sex, race, smoking, alcohol use, lifetime sexual partners, and oral oncogenic human papillomavirus (HPV) status.	Yes

**TABLE 2:** Model performance information

Study ID	Development model performance			Validation	
	AUC (95% CI) and sensitivity/specificity (where reported)	Calibration—E/O (95% CI), PPV, NPV (%)	Cut‐offs	Method of validation	Performance
Amarasinghe et al. (2010)	OPMD excl. lichen planus: 0.84 (0.81–0.87) SENS/SPEC: 93.7%/67.7% All OPMDs: 0.78 (0.75–0.81) SENS/SPEC: 81.1%/67.7%	Not reported Exc. Lichen Planus 27.5%, 98.8% (All) PPV 50.9%, NPV 89.6%	AUC of 0.87 (95% CI: 0.83–0.91), SENS 95.5%, SPEC of 75.9%	External (Different setting) Phase 2: Suburban population of the Colombo district and in a rural population in selected PHM areas of the Bulathkohupitiya MOH area in the Kegalle district of Sri Lanka.	AUC of 0.87 (95% CI: 0.83–0.91), SENS 95.5%, SPEC of 75.9%
Budhathoki et al. (Unpublished)	Not reported for development—see (internal) validation SENS/SPEC—Not reported	Plots are provided but not quantified—good calibration Not reported	Not reported	Internal The model was internally validated using a split sample approach; data was randomly split into a training (70%) and testing set (30%) Total *N* = 4601 (3030 HNC cases/1571 controls)	HNC (0.72, 95% CI = 0.69–0.75 in men and 0.75, 95% CI = 0.71–0.79 in women) (OCC—[AUC = 0.73, 95% CI = 0.69–0.77 in men and AUC = 0.79, 95% CI = 0.74–0.83 in women].) (Model + HPV serology for OPC—[AUC = 0.94, 95% CI = 0.91–0.95 in men and AUC = 0.89, 95% CI = 0.82–0.92 in women])
Chen et al. (2018)	0.768 (0.723–0.813) for men 0.700 (0.635–0.765) for women SENS/SPEC—Not reported	Plots are provided but not quantified in supplementary table. Male model had better calibration (closer to 1).	Not reported	Internal Model was internally validated with 1000 repeat samples	NA—see Development
Cheung et al. (2021)	Not reported for development—see (internal) validation SENS/SPEC—Not reported	Not reported for development—see (internal) validation Not reported	Not reported	Internal Internally validated using 5‐fold cross validation of the development population evaluated for discrimination and calibration and accounting for right censoring.	AUC = 0.84; (95% CI, 0.77–0.90) Calibration = 1.08; (95% CI, 0.81–1.44)
Koyanagi et al. (2017)	(Most extensive model) 0.72 (0.69–0.75) SENS/SPEC—Not reported	1.00 Not reported	Not reported	External Validation was carried out with a second case control study (HERPACC‐3) conducted between November 2005 and March 2013 309 head and neck cancer cases and 654 matched controls were recruited.	AUC = 0.73 (0.70–0.77) Calibration = 0.97
Krishna Rao et al. (2016)	0.866 SENS/SPEC: 0.746 (0.682–0.810), 0.846 (0.802–0.890)	Not reported PPV 0.767 (0.704–0.831), NPV 0.830 (0.785–0.875)	Development SENS—0.928 (0.890–0.966) SPEC—0.603 (0.545–0.661) PPV—0.607 (0.550–0.665) NPV—0.927 (0.888–0.965)	Internal Internally validated from original dataset with 200 bootstrap samples.	AUC = 0.865 SENS 0.744 (0.740–0.750), SPEC 0.851 (0.848–0.854) PPV 0.773 (0.768–0.777), NPV 0.830 (0.827–0.833)
Lau et al. (2018)	0.79 SENS/SPEC—Not reported	Not reported Not reported	Not reported	Internal Model underwent cross‐validation and temporal validation using a further 235 patients from same hospital	SENS/SPEC—31%, 92%
Lee et al. (2020)	Not reported for development—(see Validation) SENS/SPEC—Not reported	Provides plots for each subsite (male and female) but not quantified—mostly good calibration bar male and female hypopharyngeal cancer Not reported	Not reported	Internal Internally validated using random split sample (30%)	AUC lowest to highest = 0.643–0.820 (A) Male oral cavity cancer (AUC = 0.752); (B) Female oral cavity cancer (AUC = 0.718); (C) Male oropharyngeal cancer (AUC = 0.643); (D) Female oropharyngeal cancer (AUC = 0.745); (E) Male hypopharyngeal cancer (AUC = 0.784); (F) Female hypopharyngeal cancer (AUC = 0.820); (G) Male laryngeal cancer (AUC = 0.794); (H) Female laryngeal cancer (AUC = 0.870).
Liu et al. (2017)	1 SENS/SPEC: 100.00%, 99.02%	Not reported PPV 98.94%, NPV 100.00%	A cut‐off of 0.5 was used (50% risk)	Unclear—poor reporting 102 controls, 82 OLK, 93 OSCC	AUC = 1 SENS 100.00%, SPEC 100.00% PPV 100.00%, NPV 100.00%
McCarthy et al. (2020)	0.69 (0.66–0.71) SENS/SPEC—Not reported	Plots but not quantified for development population—CIs are also included—good calibration Not reported	Not reported	Internal Model was internally validated (split sample) with 60,240 individuals Cohort from North West England UK Biobank	Discrimination = 0.64 (0.60–0.68), Calibration = 0.83
Sharma et al. (2015)	0.9974 SENS/SPEC: 98.01%, 98.68%	Not reported PPV 99.35%, NPV 98.01%	Not reported	Internal Internally cross validated with data from original sample	(Unclear if this for same PNN/GRNN model) AUC = 0.821 SENS 87.67%, SPEC 69.46% PPV 62.86%, NPV 88.17%
Sun et al. (2019)	0.83 (0.77–0.88) SENS/SPEC: 67.53%, 81.25%	Not reported PPV 59.09%, NPV 86.19%,	Cut‐off score of 3 SENS, 67.53%, SPEC 81.25% PPV 59.09%, NPV 86.19%,	NA Development only	NA
Tikka et al. (2020) (v.2)	0.8856 (0.8818–0.8879) USOC—SENS/SPEC: 85%, 78.3% Secondary 4 week referral—SENS 97.1%, SPEC 52.9%	Not reported PPV 20.7%, NPV 98.6%.	USOC referral = 0.071 probability 4‐week 2nd clinic classification = 0.022 probability	Internal only internal validation in v.2 with 1000 bootstrap samples BUT previous version of risk model did externally validate.	See development metrics
Tota et al. (2019)	0.94; (0.92–0.97) SENS/SPEC: Not reported	1.01 (0.70–1.32) Not reported	Not reported	External External validation was conducted on a historical series of 116 oropharynx cancer cases recruited at the Johns Hopkins University	AUC = 0.87; (95% CI, 0.84–0.90) Calibration—1.08 (0.77–1.39)

A variety of cancer outcomes were considered—one model considered the risk of OPMD, and another two included the risk of developing oral cancer from OPMD. Three further models evaluated the risk of oral cancer. Six models evaluated the overall risk of HNC, two of these stratifying risk by sex and various subsites including cancers of the oral cavity, hypopharynx, oropharynx and larynx. One model considered the risk of oral and oropharyngeal cancer. Finally, one model considered the risk of oropharyngeal cancer.

Eight of the 14 models were deemed likely to require clinician input to be used, and six could possibly be used in a self‐assessment role by patients.

### 3.2 Discrimination

Discrimination, the ability of a model to discern between a positive and a negative result for disease, is a crucial performance metric of a risk model. All 14 models provided measurements of discriminatory accuracy in either their development, validation populations, or both. Ten of these models described the statistical uncertainty of their findings. Many models (*n* = 9) reported AUC values (and intervals where reported) greater than 0.7, achieving “acceptable” or “excellent” discrimination. One model with the highest AUC value reportedly achieved a “perfect” model discrimination of 1.0, however, this model was constructed from a very small sample size, in addition to other key limitations and bias concerns such as a failure to report statistical uncertainty and any missing data.^36^

### 3.3 Accuracy

Measurements of accuracy in the form of sensitivity and specificity were described in seven of the 14 studies. These ranged from 67.53% to 100%,^36^, ^39^ and 67.7% to 100% for each measure respectively.^28^, ^36^ The model that reported the highest sensitivity and specificity achieved 100% in both of these metrics in their validation population.^36^

### 3.4 Calibration

Calibration, the degree of correspondence between the estimated probability of an outcome predicted by a model vs the outcome observed is an important measurement to consider when evaluating model performance, to minimize overfitting. Despite this, calibration is often overlooked in favor of model discrimination (AUC also known as the C‐statistic).^25^ Measurements of model calibration, either in the form of an expected/observed ratio or calibration plot were described in seven out of the 14 models, however, statistical uncertainty was reported in only three of these. Calibration was evaluated in light of hierarchy definitions described by Van Calster and colleagues.^42^ Calibration was good in most, if not all, of the models where this metric was reported, with the exception of male and female hypopharyngeal cancer models in one study where calibration was sub‐optimal in these particular calibration plots.^35^ Most of the models that did report calibration presented graphs or statistics that were close to the ideal calibration slope (expected/observed) value of 1, with some models slightly above this value indicating some over‐prediction.^24^

### 3.5 PPV/NPV

PPV and NPV are defined as the proportion of patients who actually have the disease that test positive and the proportion of patients without the disease that test negative respectively. Six models reported measurements of PPV and NPV.^43^ As such only one model reported the statistical uncertainty of this.^33^ The PPV and NPV values reported ranged from 20.7% to 100% and 83% to 100%. Again, Liu and colleagues^36^ achieved the highest PPV and NPV values of 100%.

### 3.6 Model risk cut‐offs

Of the 14 models, only five reported model risk cut‐offs during development. Two models used a risk probability as a cut‐off.^36^, ^40^ Three models reported cut‐offs using performance metrics including AUC, sensitivity and specificity, and PPV/NPV.^28^, ^33^, ^39^

### 3.7 Validation

Only 3 of the 14 models reported external validation—Amarasinghe et al.,^28^ Koyanagi et al.,^32^ and Tota et al.^41^ Three others reported robust methods of internal validation via split random sampling.^29^, ^35^, ^37^

### 3.8 Risk factors

Altogether, the 14 models considered over 30 various risk factors. The most common factors included were age (13 models), alcohol consumption (13 models), sex (12 models), and tobacco smoking (12 models). Notably, two models considered HPV serostatus in model development—Budhathoki et al.^29^ and Tota et al.^41^ The number of risk factors included in models ranged between 5^36^, ^39^ and 13.^30^

### 3.9 PROBAST

The evaluation of each domain of the PROBAST risk of bias assessment tool is summarized in Table 3. Of the 14 models, seven were deemed to have a “high” risk of bias in at least one domain. The “analysis” section was the most common domain where a high risk of bias was identified. Common reasons for these included low numbers of the outcome of interest, a lack of external validation and limited or no internal validation, failure to report statistical uncertainty of findings and no discussion of missing data (and procedures in the event of this). Five of the 14 models were reported to have an “unclear” applicability concern whereby aspects of the model may limit its applicability but as such these were not major limitations. The “participants” section was the most common domain where applicability concerns were classified as “unclear.” Reasons for these included limited generalizability owing to the outcome considered, limiting the analysis to those of one ethnicity, use of non‐primary HNC cancer sites and low‐quality reporting of methods. Where models had a limitation but were otherwise fairly robust and well‐developed the risk of bias was deemed as “low.” Overall, 7 of the 14 models were deemed to have an overall low risk of bias.^28^, ^29^, ^32^, ^35^, ^37^, ^40^, ^41^

**TABLE 3:** PROBAST performance by model

Study ID	Participants		Predictors		Outcome		Analysis	Overall	
	Risk of bias	Applicability concern	Risk of bias	Applicability concern	Risk of bias	Applicability concern	Risk of bias	Risk of bias	Applicability concern
Amarasinghe et al. (2010)^28^	LOW	UNCLEAR	LOW	LOW	LOW	LOW	LOW	LOW	UNCLEAR
Budhathoki et al. (Unpublished)^29^	LOW	UNCLEAR	LOW	LOW	LOW	UNCLEAR	LOW	LOW	UNCLEAR
Chen et al. (2017)^30^	LOW	LOW	LOW	UNCLEAR	LOW	LOW	HIGH	HIGH	LOW
Cheung et al. (2021)^31^	LOW	LOW	LOW	LOW	LOW	LOW	HIGH	HIGH	LOW
Koyanagi et al. (2017)^32^	LOW	LOW	LOW	LOW	LOW	LOW	LOW	LOW	LOW
Krishna Rao et al. (2016)^33^	UNCLEAR	LOW	LOW	LOW	LOW	LOW	HIGH	HIGH	LOW
Lau et al. (2018)^34^	UNCLEAR	LOW	UNCLEAR	LOW	UNCLEAR	LOW	HIGH	HIGH	LOW
Lee et al. (2020)^35^	LOW	LOW	LOW	LOW	LOW	LOW	LOW	LOW	LOW
Liu et al. (2017)^36^	HIGH	LOW	UNCLEAR	UNCLEAR	LOW	LOW	HIGH	HIGH	UNCLEAR
McCarthy et al. (2020)^37^	LOW	LOW	LOW	LOW	UNCLEAR	LOW	LOW	LOW	LOW
Sharma et al. (2015)^38^	HIGH	UNCLEAR	HIGH	UNCLEAR	HIGH	UNCLEAR	HIGH	HIGH	UNCLEAR
Sun et al. (2019)^39^	HIGH	UNCLEAR	LOW	LOW	UNCLEAR	LOW	HIGH	HIGH	UNCLEAR
Tikka et al. (2020) (v.2)^40^	LOW	LOW	LOW	LOW	LOW	LOW	LOW	LOW	LOW
Tota et al. (2019)^41^	LOW	LOW	LOW	LOW	LOW	LOW	UNCLEAR	LOW	LOW

### 3.10 Overall quality assessment

The overall quality assessment of the 14 models and the components considered in this quality assessment along with model predictive performance assessment can be seen in Table 4. Of the 14 models, six were assessed as “high” quality,^28^, ^32^, ^35^, ^37^, ^40^, ^41^ three as “moderate” quality, and five as “low” quality. The main components, which impacted on quality were PROBAST risk of bias, applicability concern, and validation methods.

**TABLE 4:** Overall quality/performance assessment

Study ID	Quality				Performance
	PROBAST bias	PROBAST applicability	Validation—external, internal, no	Overall quality assessment	AUROC
Amarasinghe et al. (2010)^28^	LOW	UNCLEAR	External	HIGH	0.87 (95% CI: 0.83–0.91)
Budhathoki et al. (Unpublished)^29^	LOW	UNCLEAR	Internal—large data 70/30 split	MODERATE	HNC (0.72, 95% CI = 0.69–0.75 in men and 0.75, 95% CI = 0.71–0.79 in women)
Chen et al. (2018)^30^	HIGH	LOW	Internal—bootstrap	MODERATE	0.768 (0.723–0.813) for men, 0.700 (0.635–0.765) for women
Cheung et al. (2021)^31^	HIGH	LOW	(RCT design), Internal—cross validation	MODERATE	0.84; (95% CI, 0.77–0.90)
Koyanagi et al. (2017)^32^	LOW	LOW	External	HIGH	0.73 (0.70–0.77)
Krishna Rao et al. (2016)^33^	HIGH	LOW	Internal—bootstrap	LOW	0.865
Lau et al. (2018)^34^	HIGH	LOW	Internal—cross and split validation	LOW	0.79
Lee et al. (2020)^35^	LOW	LOW	Internal—large data 70/30 split	HIGH	Poorest model to best = 0.643–0.820
Liu et al. (2017)^36^	HIGH	UNCLEAR	Unclear	LOW	1
McCarthy et al. (2020)^37^	LOW	LOW	Internal—large data split	HIGH	0.64 (0.60–0.68)
Sharma et al. (2015)^38^	HIGH	UNCLEAR	Internal—cross validation	LOW	0.9974
Sun et al. (2019)^39^	HIGH	UNCLEAR	No	LOW	0.83 (0.77–0.88)
Tikka et al. (2020) (v.2)^40^	LOW	LOW	Internal—bootstrap, BUT previous version did	HIGH	0.8856 (0.8818–0.8879)
Tota et al. (2019)^41^	LOW	LOW	External	HIGH	0.87 (0.84–0.90)

In terms of performance, eight of the 14 models were high performing, with AUCs greater than 0.8, ranging from 0.83 to 1.^28^, ^31^, ^33^, ^36^, ^38^, ^39^, ^40^, ^41^ Of the six high quality models, three had high predictive performance with good discriminative accuracy—Amarasinghe et al.,^28^ Tikka et al.,^40^ and Tota et al.^41^

### 3.11 Synthesis

All six of the high‐quality models were more recently developed (since 2010). Despite the heterogenicity of the models, generally, those that were assessed as high quality shared common design aspects. All of the models were developed from case–control study data with some variation in design such as hospital‐, community‐, synthetic‐controls, or a mixture of population and hospital controls. All six high quality studies also used a form of logistic regression to derive their risk models. These included binary, multivariate or conditional logistic regression. Three of the models required clinician input to use: two of these due to HPV or genotype information,^32^, ^41^ and one due to use of clinical examination information.^40^

With regards to factors included in the high‐quality models, all six had some sociodemographic factors—age was evaluated in all six of them and sex in five—one model did not analyze or adjust for sex which in turn resulted in reduced applicability. Four of the models adjusted for at least one additional sociodemographic factor (education, ethnicity, or socioeconomic deprivation).^28^, ^35^, ^37^, ^41^ Two high‐quality models evaluated socioeconomic deprivation, one synthesizing educational and occupational status to define this, the other measured deprivation using an area based socioeconomic index.^28^, ^37^ Similarly, all six also incorporated behavioral factors into their model—using both alcohol intake and smoking. Notably, one model used betel quid chewing as an additional behavioral predictor, an important aetiological risk factor for the target population for this model.^28^ One model also used exercise and fruit/vegetable intake as additional factors.^37^ Two of the three models that used biomarker (genetic or HPV) data in their models were ultimately among the six assessed as high quality. One high quality model used HPV serostatus as a predictor.^41^ Another used DNA sampling to assess *ALDH2* genotype.^32^ Only one of the high‐quality models reported family history as a predictor.^35^ Finally, one of the six high quality models used clinical signs and symptoms as predictors to inform model design.^40^

The critical design feature common to all of the high‐quality models was robust validation methods. These included the use of external validation in another setting,^28^, ^32^, ^41^ a history of this (in a previous developmental version of the model),^40^ or the utilization of well‐conducted internal validation with a large split sample.^35^, ^37^ All the high‐quality models that did not use an external validation approach described this as a limitation and a next step in their model development.

In addition to the high quality models having a low risk of bias, five of the six also had a low applicability concern,^32^, ^35^, ^37^, ^40^, ^41^ while the remaining model scored “unclear” for applicability concern assessment.^28^ This was primarily due to the model evaluating the risk of OPMD only and not accounting for sex as a predictor during model development.

The high‐quality models mostly had fair to good performance. One model had a sup‐optimal AUC of 0.64,^37^ two high quality models reported a fair AUC over 0.7^32^, ^35^ and three of the high quality models achieved excellent AUC values over 0.8.^28^, ^40^, ^41^ The better the discriminative performance, the more accurately those at risk of disease can be identified. Two of the six high quality models did not report calibration metrics.^28^, ^40^ This was the main limitation of these models. Four models reported calibration, either numerically or via plots, illustrating that the models could accurately predict outcomes in line with the observed event of interest (HNC), with most of the calibration graphs or reported expected/observed values being close to the perfect prediction value of 1. Therefore, for the most part, HNC models, where reported, showed good calibration between the expected and observed risk of HNC.

Of the six high quality models, three were also high performing, achieving excellent discrimination with AUCs over 0.8.^28^, ^40^, ^41^ The three models predicted the risk of OPMD, HNC, and oropharyngeal cancer respectively. Two of the models were externally validated in another cohort,^28^, ^41^ while the other model had a history of external validation in its first version.^40^ This model has also seen some clinical use, being used to triage patients remotely during the COVID‐19 pandemic.^44^ The three high‐quality, high performing models all used similar sociodemographic and behavioral factors but where the three high performing models differed and ultimately excelled was in the choice of additional predictors used. These included the aforementioned use of betel quid in one model,^28^ clinical examination and symptoms in another model,^40^ and the use of HPV serostatus and ethnicity in the third high quality model.^41^

Those that were classified as moderate overall quality had at least one major methodological limitation, but generally had fair predictive performance. Studies that were classified as low quality had at least two significant limitations; some of these reported very good model discrimination, although this needs to be interpreted with caution.

## 4 DISCUSSION

A range of risk prediction models for HNC were identified. These models were heterogeneous in their risk factors and outcomes, were developed with variable methodological approaches and rigor, and several models demonstrated the potential to predict and identify those at a higher risk of HNC.

The six high performing models incorporate the major HNC risk factors of tobacco smoking and alcohol consumption, in addition to the sociodemographic factors of age and sex. Additional factors are included contributing to improved performance. Four included socioeconomic factors, one: family history, one: betel quid chewing, one: HPV serology, one included a genetic marker, and one included clinical examination findings. These selected factors are consistent with the international analytical epidemiological literature which has identified tobacco smoking and alcohol drinking as the major risk factors (accounting for up to ~70% of the population attributable risk),^45^, ^46^, ^47^ the important role of demographics of age in cancer risk,^48^ and sex—particularly men being more predisposed to HNC.^49^ Moreover, the important role of HPV particularly in oropharyngeal cancer^50^ and betel quid chewing in oral cavity cancer in particular populations,^51^ and the increasingly refined role of genetic factors in HNC are reflected in the models.^52^, ^53^ Three of these high performing predictive models were of high quality and consistent methodological rigor—all included major behavioral and sociodemographic factors along with an additional factor. They were generally more specified models that were tailored to their target population or subsite, for example, to South Asia (the inclusion of betel quid)^28^ or to oropharyngeal cancer (the inclusion of HPV serology).^41^ Perhaps counterintuitively, some of the models that included many additional risk factors generally had lower predictive performance. This could be explained by a statistical phenomenon known as model overfitting, whereby a model becomes too tailored to a developmental dataset with unnecessary components. This violates the principle of parsimony, in turn limiting a model's generalisability when applied to another independent dataset.^54^ The higher performing models and particularly the high performing, high‐quality models generally required clinician input, reflecting the nature of the variables required.

It could be hypothesized that HPV serostatus and genetic markers could help better inform individual risk, in line with the growing popularity of “personalized medicine” in other diseases. However, this may in turn limit the practicality of a model—for example, primary care medical or dental practices where time and resources may already be limited.

Several similar reviews of risk prediction models have been undertaken for other cancer sites including colorectal^55^, ^56^, ^57^, ^58^ and lung.^59^ The reviews of colorectal and lung cancer models both identified models with high performance (0.65–0.75, 0.76–0.96, and 0.57–0.879), while the breast cancer models generally reported poorer performance (0.56–0.63 and 0.56–0.71). The poor performance of these breast cancer models was attributed in the reviews to limited knowledge and data on risk factors for breast cancer leading to sub‐optimal prediction. The performance range of the HNC models was similar to the colorectal and lung cancer reviews and might in part be attributed to the growing epidemiological research base in the field.^60^, ^61^

The methodology of this review is similar to reviews of risk models for other cancers.^55^, ^56^, ^57^, ^58^, ^59^ This review employed robust quality and methodological assessment including the PROBAST risk of bias and applicability concerns tool, and a focus on the nature of model development and validation approach. While validation is a domain of the PROBAST tool, this was explicitly assessed separately as external validation is gold standard methodology of risk prediction model development.^62^, ^63^ To our knowledge, this is the first review of HNC risk prediction models. This review has some strengths including searching multiple databases, dual article screening, as well as the comprehensive quality assessment. A detailed thematic narrative synthesis drew on the model quality and performance to identify key design characteristics.

There are some limitations to this review, including not publishing a protocol. The study started as a rapid review—but ultimately became more systematic in nature—particularly in term of quality assessment methods. However, it was not feasible to register the review retrospectively, hence the review was not registered with PROSPERO. The PICO/research question and search/inclusion criteria were developed a priori and did not change during the review. The review was also conducted following PRIMSA guidelines and was advised by a subject librarian in the field. Second, the inclusion of papers published only in English may have excluded other pre‐existing models. As with most reviews, the nature and limitations of available data can influence the overall quality of evidence synthesized—the source data of this review are largely from case–control studies which do have some potential recall and selection biases.^64^

This review has also been conducted within the overarching objectives of developing a risk model for HNC and translating it to a clinical setting. Any findings of this review are intended to help inform model development.

## 5 CONCLUSIONS

This review illustrates that there is a limited but growing number of HNC risk prediction models. Some of the models reviewed do have the potential to identify and stratify those at risk of HNC. Model predictor selection should include, as a minimum, well established risk factors as well as sociodemographic predictors. Additional genetic, biomarker, or clinical factors have the potential to improve predictive performance. However, care should be taken to ensure a limited number of predicting factors are chosen to avoid model overfitting. Such early identification of risk factors in the context of a HNC risk level could have important applications including using this “teachable moment” for behavior change, directing patients to preventive care pathways (e.g., for smoking cessation), or in identifying the need for tailored frequencies in recall intervals for clinical examination (e.g., with primary care dental practitioners). These models could form the basis of a personalized approach to HNC prevention. Further work can be undertaken to refine, improve and validate these models and potentially trial in the clinical setting.

## CONFLICT OF INTEREST

David I. Conway is associated with two of the publications in this review: David I. Conway is a Glasgow center PI on the ARCAGE multicenter study and is a co‐author of one the models developed from this study (submitted)^29^; David I. Conway is also a member of the INHANCE Consortium from which another model was developed.^35^ All other authors have none to declare.

## Supporting information


**Supplementary Material** Search term list and results
