# Supplementary material for: Risk prediction models for head and neck cancer: A rapid review
Source: Laryngoscope Investig Otolaryngol. 2022 Nov 28;7(6):1893–908. doi: 10.1002/lio2.982 (PMC9764804; doi:10.1002/lio2.982)
Supplement: Supplementary file 1 — Supplementary Material Search term list and results [file LIO2-7-1893-s001.docx]

**Supplementary Material**

(((risk prediction or risk factor or risk model or risk assessment or risk calculator or risk tool or risk score) and (cancer* or tumour or tumor or neoplas* or malignan* or squamous cell carcinoma) and ((head and neck) or oral cancer or oral cavity cancer or oropharynx cancer or oropharyngeal cancer or larynx cancer or laryngeal cancer leukoplakia or erythroplakia or submucous fibrosis or OPMD or Oral Potentially Malignant Disorder)) not (prognos* or survival))

Abstract + title (tw), no filters

Embase: 1787 results 22:03 23/09/2021

Medline: 1258 results 22:03 23/09/2021
